# ‘Is It Safe? Is it not?’ A Youth‐Led Photovoice Study of Youth Perspectives of COVID‐19 Vaccine Confidence

**DOI:** 10.1111/hex.70051

**Published:** 2024-10-05

**Authors:** Shelby Mckee, Natasha Y. Sheikhan, Sean Patenaude, Jo Henderson, Rodney Knight, Sean A. Kidd, Skye Barbic, Aileen O'Reilly, Lisa D. Hawke

**Affiliations:** ^1^ Centre for Addiction and Mental Health Toronto Ontario Canada; ^2^ University of Toronto Toronto Ontario Canada; ^3^ Université de Montréal Montréal Québec Canada; ^4^ University of British Columbia Vancouver British Columbia Canada; ^5^ Alone Dublin Ireland

**Keywords:** co‐development, COVID‐19, engagement, mental health, photovoice, substance use, vaccine confidence, vaccine hesitancy, youth

## Abstract

**Background:**

Youth have been uniquely affected by the COVID‐19 pandemic. Despite high rates of COVID‐19 infection, youth had one of the lowest vaccine uptake rates. Certain characteristics can affect vaccine uptake, such as mental health and substance use, but it is important to understand uptake for an effective response to pandemics.

**Objective:**

This study examined the perspectives of youth with mental health or substance use concerns on COVID‐19 vaccine confidence, hesitancy and overall COVID‐19 vaccine perspectives.

**Methods:**

Using photovoice, a community‐based participatory research method, a sample of 27 youth aged 14−24 years participated in a series of photography workshops and focus groups. Participants submitted final photographs for discussion. Focus groups were recorded, transcribed and thematically analysed.

**Results:**

Four themes were generated: (1) Youth deciphered the vaccine discourse in a changing information landscape; (2) mixed perspectives of families, friends and loved ones influenced the vaccine journey; (3) complex societal influences affected views and decisions around the COVID‐19 vaccine; and (4) youth navigated their vaccine journeys through first‐ and second‐hand experiences. The four themes and subthemes highlight the evolution of youth's journeys with the COVID‐19 vaccine over the course of the pandemic and into the late‐pandemic period.

**Conclusions:**

Youth with mental health or substance use challenges navigated a complex environment during the COVID‐19 pandemic. The wide variety of factors influencing vaccine perspectives should be taken into account in public health messaging and future research on youth vaccine uptake. Youth‐led and youth‐engaged research can help solicit rich and meaningful perspectives of young people on important public health issues.

**Patient or Public Contribution:**

This was a youth‐led study. A youth research analyst conducted the study activities together with the support of a youth advisory group, an adult photographer with lived experience, and a scientific team.

##  

The COVID‐19 pandemic caused worldwide morbidity and mortality, challenging daily life and overall well‐being [1]. The production and distribution of a vaccine have been key to protecting the population, reducing infection rates and supporting a socioeconomic and personal recovery [2, 3]. Many countries, including Canada, adopted vaccine prioritization as a targeted solution; however, positive public attitudes are essential for vaccination success as a public health strategy [2, 4]. In the case of the COVID‐19 vaccine, the rapid development, new vaccine formula and complexity of the pandemic led to public confusion and a sense of uncertainty about the vaccine for many [5, 6, 7].

The United Nations defines youth as people aged 15−24 years [8]. Youth are at a key developmental period, marked by developmental milestones such as establishing autonomy, social relationships and transitions in education occupation [9, 10]. This developmental period is concurrent with brain and behavioural control maturation [11]. During this time, youth are particularly vulnerable to psychological distress, as they may not have as yet developed the tools to deal with life stressors [9, 12].

The measures implemented in response to the COVID‐19 pandemic disrupted developmental milestones for youth [13, 14]. Numerous studies have found increased stress, anxiety, depression, trauma‐related distress and a negative mental health impact early in the pandemic [13, 15, 16, 17]. Youth with preexisting vulnerabilities, such as mental health and/or substance use (MHSU), lower socioeconomic conditions, family instability and gender diversity, may have been at particular risk [14, 15, 18].

Vaccine hesitancy refers to delaying vaccination or not getting vaccinated when vaccines are available [19]. The WHO Strategic Advisory Group of Experts [20] proposed the ‘3 Cs’ model to understand the complexities of vaccine hesitancy and promote vaccine confidence [20]. The ‘3 Cs’ include (1) *confidence* (trust/mistrust in the vaccine), (2) c*omplacency* (perception of risk and need) and (3) *convenience* (how easy the vaccine is to access). Recent research on the COVID‐19 vaccine has proposed a ‘4 Cs’ model, with (4) *communications*, which refers to messaging and measures surrounding the vaccine [21]. These models underpin complex decision‐making that is influenced by an individual's context, personal and group situation and vaccine‐specific factors; perspectives and decision‐making are further determined by a range of cognitive, emotional, psychological, cultural, social, spiritual and political factors that vary across vaccine, place and time [19, 20, 22]. This model provides a framework for understanding vaccine uptake across a broad number of actionable factors.

Across ages, youth had the highest COVID‐19 infection rate, and the lowest vaccination uptake rates [23]. The perception of COVID‐19 infection risk may contribute to low vaccination rates in youth [24]. Higher levels of COVID‐19 vaccine hesitancy in youth have been associated with being female, having lower education, using social media as an information source, having an unfavourable attitude and having poor knowledge about COVID‐19 and the vaccine [25, 26]. Sociodemographic characteristics, COVID‐19 knowledge and history of adversity influence willingness to get vaccinated among youth with MHSU [27, 28]. One study found that youth with MHSU challenges had low vaccine hesitancy early in the pandemic, but higher vaccine hesitancy later in the pandemic, demonstrating the interaction between mental health and vaccine attitudes [29].

Developing public health messaging for and with vulnerable communities is key to optimizing vaccine uptake [30, 31] and better preparing mitigating strategies for vaccine hesitancy in the future [32, 33, 34]. Participatory research such as photovoice is particularly well suited to understanding sensitive, controversial or stigmatizing issues [35]. Early research has demonstrated that participatory research and photovoice can highlight the unique perspectives and experiences of youth around the COVID‐19 pandemic [28].

## 1 Objective

1.1

This photovoice study aimed to understand the perspectives of youth with mental health and/or substance use challenges on COVID‐19 vaccine confidence and hesitancy.

## 2 Methods

This qualitative descriptive study used a contextualist epistemology that highlights the rich knowledge that youth generate through their experience, embedded in their local and personal contexts [36, 37]. We used photovoice to co‐create knowledge about youth perspectives on COVID‐19 vaccines. Photovoice is a community‐based participatory research methodology that uses expressive imagery and group discussion, stimulating critical thinking and open dialogue to contextualize personal and community strengths and weaknesses surrounding a topic [38]. It draws on the importance of individual voice and documentary photography to empower individuals to promote social change [39]. This study was guided by recommendations of the Canadian Institutes of Health Research (CIHR) Strategy for Patient‐Oriented Research (SPOR) [40]. It was led at the Centre for Addiction and Mental Health (CAMH) and approved by the CAMH Research Ethics Board (Approval no. #155‐2021).

### 2.1 Participants and Recruitment

The sample consisted of 27 youth aged 14−24 years who self‐reported MHSU during the pandemic and who reported current or past vaccine hesitancy. We recruited from multiple sources: (1) direct outreach to partners and contacts from youth organizations (four participants recruited); (2) purposive sampling from preexisting research databases of participants who consented to be contacted for future research (two participants recruited); and (3) advertising on social media platforms (23 participants recruited; six participants recruited from other or unknown sources). Of these, 27 completed the project. We had multiple recruitment flyers that were co‐created with our youth advisory group. A standard recruitment flyer was used for our first two recruitment sources. An additional seven brief Facebook and Instagram ads were used. All our recruitment flyers emphasized the project's goal of exploring the perspectives of youth with mental health or substance use challenges regarding COVID‐19 vaccine confidence and hesitancy using photovoice. Potential participants reached out to the youth research analyst (RA) for more information. Recruitment continued until the desired sample size was reached and all consenting youth had been offered the opportunity to participate. We aimed for a larger sample than most photovoice projects to address a current limitation in photovoice research [41].

### 2.2 Procedure

All research activities were completed virtually. Participants individually attended an informed consent discussion on WebEx teleconferencing software; they completed written informed consent and a demographic questionnaire on the REDCap electronic data capture software [42]. Youth participated in seven 1 h workshops and one 2 h focus group, via WebEx, between January and May 2023. Workshops focused on participants' COVID‐19 vaccine experiences while building their skills as photographers, combined with critical thinking. Workshop topics included ethics, photography techniques, photo editing, annotating and photo sharing. We also allowed for flexibility in content to allow participants a sense of ownership and freedom to implement their own ideas. Throughout the workshops, optional activities were utilized to help participants explore their COVID‐19 vaccine perspectives. Activities were co‐designed and focused on topics such as home, safety, community, and so forth. The probe for the final photo(s) was general, around COVID‐19 vaccine confidence, to avoid dictating the content of photographs [43].

Following the workshops, participants submitted their final photographs to discuss during the focus groups. Participants did not have to submit a final photo to participate in the focus group; however, all except one did. Participants received a $25 e‐gift card for each attended workshop, a $50 e‐gift card for the focus group and a $50 e‐gift card upon submission of a final photo. Participants were allowed a maximum of three photos for their final photo submission, with a total of 58 final photos being submitted from 26 participants. There were 8−10 participants per workshop and 3−5 participants per focus group. Focus groups and workshops were co‐facilitated by the youth RA and another research staff or a photographer with MHSU lived experience.

### 2.3 Measures

The demographic characteristics collected included age, gender identity, geographical region, ethnic background, level of schooling, employment status and other variables about COVID‐19 vaccination status. The semi‐structured focus group guide asked participants to reflect on their photographs and elicit dialogue about COVID‐19 vaccine perspectives. Focus groups were audio‐recorded, transcribed by a professional transcription agency, de‐identified, and verified for accuracy by the youth RA. The focus group guide is provided in Appendix SA.

### 2.4 Lived Experience Engagement

This study was designed to be youth‐led. A youth RA with MHSU lived experience conducted the study activities. She was supported by a 4‐member youth advisory group, with two youth engagement specialists during the early stages of the study. Advisory group membership was refreshed at the data analysis stage, leading to a new 6‐member youth advisory group. One lived experience adult photographer also supported the project. Experienced researchers supported the youth throughout the study in methodological, operational and analytical processes, but encouraged the youth to make key decisions.

### 2.5 Data Analysis

Focus group data were analysed inductively using Clarke, Braun, Hayfield [44] reflexive thematic analysis. Thematic analysis is an iterative process that follows multiple stages [45]: (1) familiarization; (2) generating initial codes; (3) searching for themes; (4) reviewing themes; (5) defining and naming themes; and (6) producing the report [44]. Transcripts were uploaded to NVivo 14 [46]. The youth RA identified and collated the data relevant to each code, with regular consultations with study team members. Codes were arranged into potential themes and subthemes. We reviewed and verified the consistency of the codes within themes. A detailed analysis was conducted to minimize thematic overlap and describe each theme's contribution to the story in the data set. Themes are illustrated with representative quotes, some of which were selected together with the youth advisory group members. The photographs herein were chosen together with the youth advisory group to accurately represent each theme.

### 2.6 Positionality

The youth RA is a young adult with MHSU lived experience and a bachelor's level of training. She is fully vaccinated, has received a booster and plans on getting more in the future. She has confidence that vaccination was needed to protect herself and others, as well as essential for public health; however, she did experience her own levels of COVID‐19 vaccine hesitancy. The research lead is a PhD‐level scientist with a background in psychology; she has conducted substantial youth‐related research and COVID‐19 research. Although she has maintained a stance of vaccine confidence, the participants' dialogues of vaccine hesitancy, concern and information overload resonated with her experience of the pandemic.

## 3 Results

Participant characteristics are described in Table 1. Participants were relatively diverse across age, ethnicity, location and other factors. The average age was 19.6 years (SD = 3.1). Participants attended M = 6.3 workshops (SD = 0.9, range = 4−7). The majority were girls/young women, aged 18−24 years, from large urban centres and from Central Canada. Although the majority of participants were fully vaccinated, less than half reported having received all COVID‐19 vaccines available to them as soon as they were available.

**Table 1:** Sociodemographic characteristics of participants.

Demographic characteristics (*N* = 27)	*n* (%)
Age	
< 18	10 (37.0)
18−24	17 (63.0)
Gender identity	
Boy/man	4 (14.8)
Girl/woman	22 (81.5)
Nonbinary or transgender	1 (3.7)
Ethnicity	
White	6 (22.2)
East or Southeast Asia	9 (33.3)
South Asian	5 (18.5)
Black	2 (7.4)
More than one ethnicity	5 (18.5)
Location of residence	
Western Canada	10 (37.0)
Central Canada	14 (51.9)
Eastern Canada	3 (11.1)
Community size	
Large urban centre	15 (55.6)
Medium population centre	7 (25.9)
Small centre or rural area	5 (18.5)
Employment status	
Employed	13 (48.1)
Unemployed	13 (48.1)
Other/missing	1 (3.7)
Student—yes	21 (77.8)
Born in Canada—yes	21 (77.8)
Self‐reported physical health	
Good to excellent	23 (85.2)
Fair to poor	4 (14.8)
Self‐reported mental health	
Good to excellent	15 (55.6)
Fair to poor	12 (44.4)
Received all available COVID‐19 vaccines	
Yes, immediately	11 (40.7)
Delayed vaccination, but did get them	9 (33.3)
No, has not received all vaccines	6 (22.2)
Missing/other	1 (3.7)

Four themes were generated: (1) Youth deciphered the vaccine discourse in a changing information landscape; (2) mixed perspectives of families, friends and loved ones influenced the vaccine journey; (3) complex societal influences affected views and decisions around the COVID‐19 vaccine; and (4) youth navigated their vaccine journeys through first‐ and second‐hand experiences. Specific patterns were grouped into subthemes to illustrate the progressive evolution of participants' sense of COVID‐19 vaccine confidence over the course of the pandemic. The themes and subthemes are illustrated in Table 2 and described below, with representative quotes.

**Table 2:** Summary of themes and subthemes generated from the data.

Theme	Subtheme
Youth deciphered the vaccine discourse in a changing information landscape.	Lack of reliable sources of information and hearing negative and conflicting information about the vaccine
	Increased literacy leading to confidence
	Continual perpetuation of unreliable information and new regulations leading to a resurgence of hesitancy
Mixed perspectives of families, friends and loved ones influenced the vaccine journey.	Perspectives of family and loved ones shape opinions
	Negotiating vaccine perspectives despite differences in opinions among family and friends
	Acceptance of own position in the context of social differences
Complex societal influences affected views and decisions around the COVID‐19 vaccine.	Rigid community perspectives about the vaccine as the only solution
	Pressure to get vaccinated to reengage in society and social activities
	Pro‐social motivations
	Confidence, ambivalent and regret in the aftermath of the vaccine
Youth navigated their vaccine journeys through first‐ and second‐hand experiences.	Mixed past experiences with healthcare shape early opinions
	Seeing positive and negative effects of COVID‐19 (vaccine and disease)
	Late pandemic vaccine perspectives are based on the sum of pre‐pandemic, early pandemic and mid‐pandemic experiences

### 3.1 Youth Deciphered the Vaccine Discourse in a Changing Information Landscape

**Figure 1:**
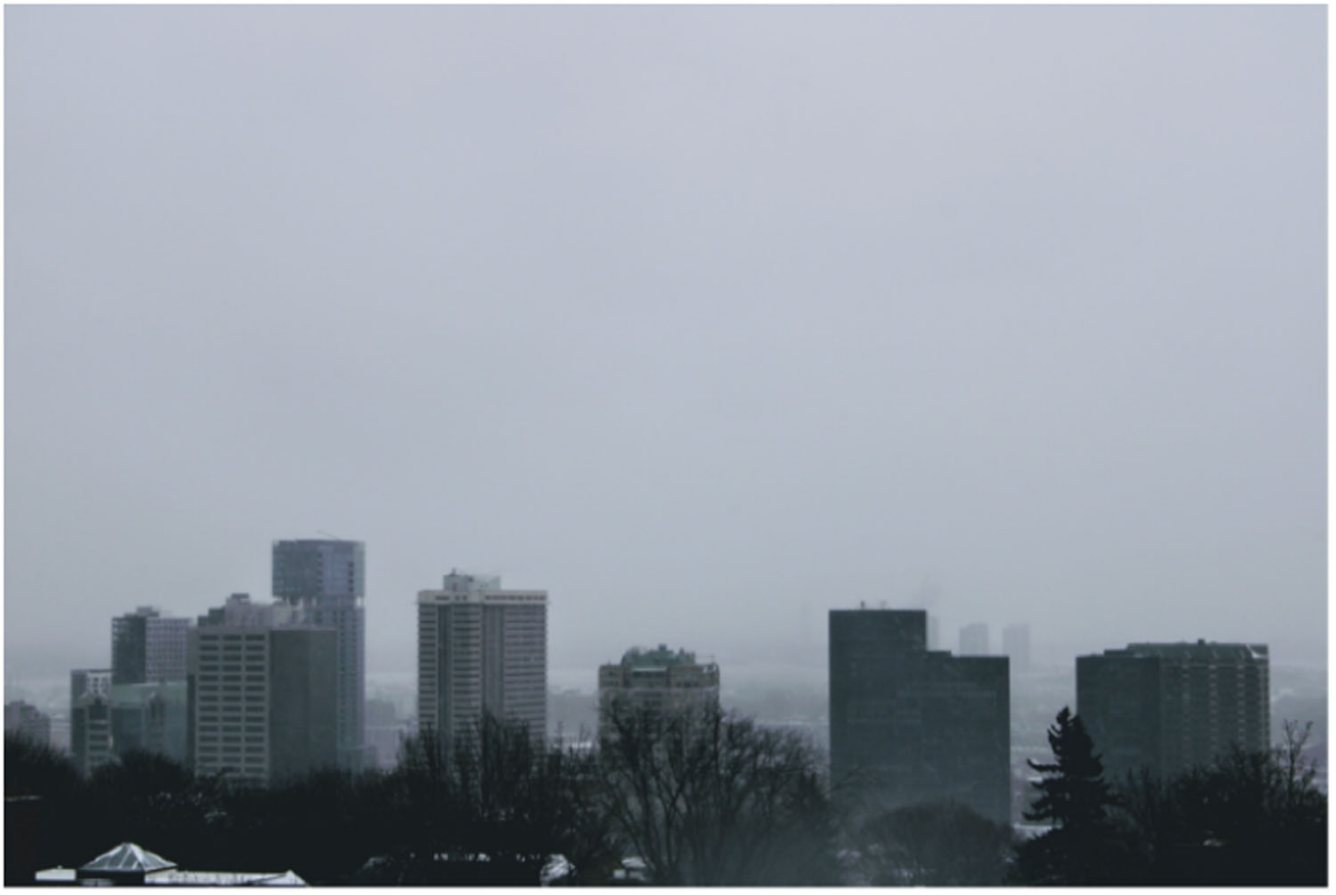
Participant photograph. ‘The death of cement: SARS‐CoV‐2. mRNA vaccine. So many notions foreign to the average citizen. Disinformation. Hatred. A TV full of bad news. How do I know what information is reliable? Supply of vaccines obtained from foreign countries. Partnerships. Financing. What if the government's goal wasn't just the health of its citizens? How should I feel? What should I believe? I am only one person, alone among these cement giants, these buildings whose use I do not know, alone among this funeral fog that blinds me, that prevents me from knowing the truth. ALL ALONE.’

Participants described that early in the pandemic, they experienced challenges understanding vaccine information in the rapidly changing information landscape. Participants noted their difficulty accessing reliable COVID‐19 vaccine information in the context of emerging and often contradictory sources. Reliable information served as an anchor for vaccine confidence, whereas unreliable information eroded that confidence over time (see Figure 1).

Participants emphasized that the volume of conflicting information from social media and news reports contributed to the initial experience of vaccine hesitancy. They felt that information was highly polarized, that is, positive and pro‐vaccine or negative and anti‐vaccine, and they struggled to decipher what was right from wrong, which made it difficult to ‘decide what is wrong and what is right and come to a conclusion on whether to vaccinate’*.*

Several discussions focused on challenges with inconsistent vaccine information and dealing with the complexities of COVID‐19 as a disease. Participants' ability to interpret vaccine recommendations was blurred by the contradictory information and indigestibility of the science. For instance, one participant noted, ‘with my limited scientific knowledge, it almost felt [like] so much work, so much anxiety, trying to be like, is this valid? How do I fact‐proof this, you know?’

Participants discussed information sources, stating that the vaccine was developed too quickly, that there were insufficient data on the COVID‐19 virus and that there were issues with vaccine technology. Information supporting possible compromises in the safety and quality of the vaccine contributed to hesitancy and mistrust: ‘We were in a rush to get the vaccine. But it got developed so fast. Usually it takes a while. So, it was kind of like, you know, “Is it safe? Is it not?” I don't think the government would give us something unsafe, but you never know.’ Information sources have the ability to instil fear regarding short‐ and long‐term side effects, including topics of conspiracy: 'just overall that fear of being tracked, as well as the fear of what exactly is going into our bodies.’

Participants talked about the negative emotions that they felt towards the constant stream of information and how it affected their mental health. One participant noted, ‘The feeling of being overwhelmed and just feeling almost anxiety‐inducing (…). Just going through news—every day seemed like breaking news—so it just felt really overwhelming.’

With the release of the COVID‐19 vaccine and as more information became available, participants' perspectives continued to evolve. Progress towards confidence was contingent on the consistency and credibility of information. Participants found resources through news reports, doctors, research documents and teachers. Those who had access to resources that felt they could trust were more confident in their decision to receive it.

I had a lot of vaccine hesitancy in the beginning. I didn't want to take the vaccine. But after I kind of got more educated on the benefits of the vaccine, how it works, and once I got some statistics about how many people have taken the vaccine and reviews, etcetera and the positive change it's making, it kind of encouraged me to further take the vaccine at the end.

However, some participants discussed experiencing confusion when the recommendations and information from trusted sources contrasted: ‘I've asked multiple doctors about the vaccine and something that was really difficult, is they did not all have the same answer (…) it was kind of [like] I'm trying to discern who to trust and what not to trust and making the decision for myself.’

As the pandemic progressed, conflicting and changing information continued to circulate, resulting in a resurgence in vaccine hesitancy for some. Discussions revolved around questioning the decision to be vaccinated and the validity of previous theories about the vaccine's distribution, efficacy and safety.

Then, all of the stuff with [vaccine name] came out with the blood clots and they told people not to do it. And I had got my mum vaccinated with [vaccine name] because I trusted it. And the government pulled back and said, 'No, this actually isn't safe, and everybody who had [vaccine name] should get different shots now.' And that's where I started to get confused.

There were negative reactions towards booster shots, but these were not universal. For some participants, vaccine confidence had gained a stronger ground and the booster vaccines were seen as a positive step.

### 3.2 Mixed Perspectives of Families, Friends and Loved Ones Influenced the Vaccine Journey

**Figure 2:**
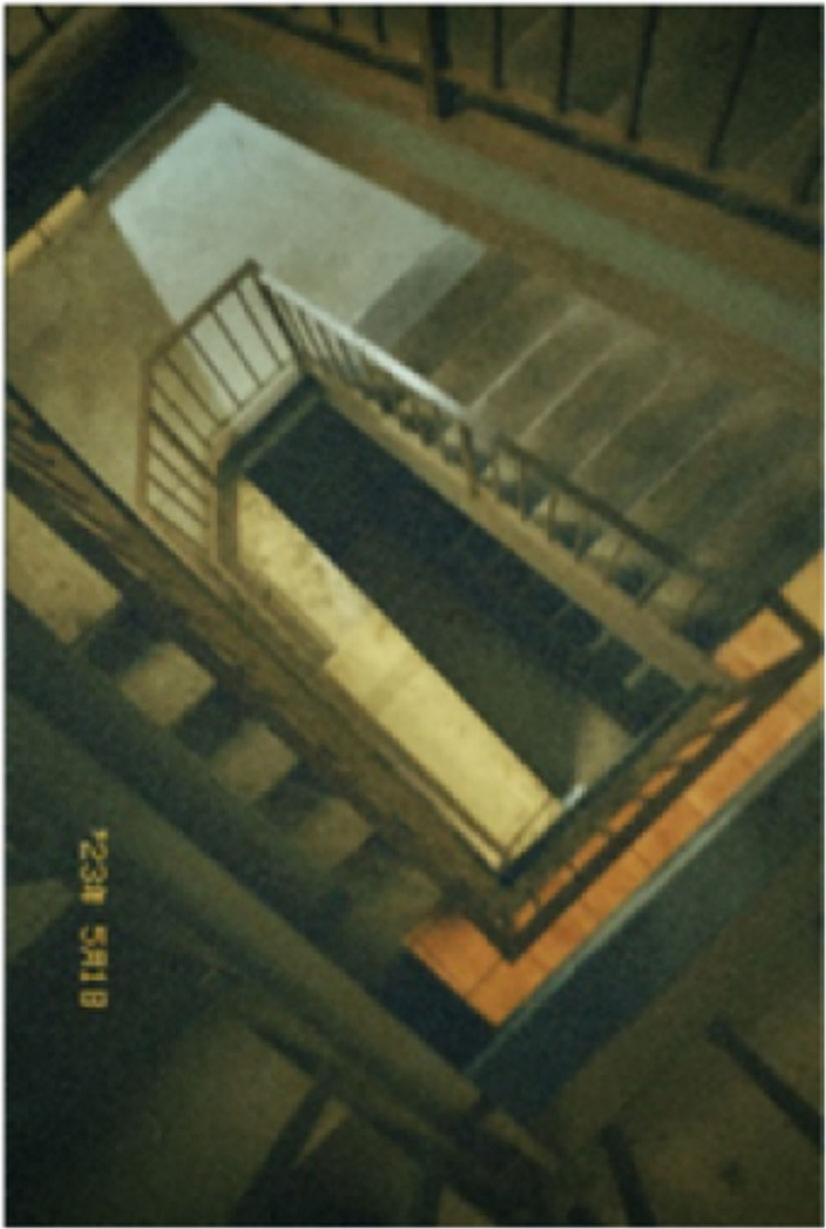
Participant photograph. ‘Fallen: I was so confused on what to do with the new COVIDvaccines because of how many different opinions were thrown at me. Ideas from online, from my family, friends and so forth. The choice to get or to not get the vaccine made me feel lost‐like – I've fallen down an infinite loop and couldn't see the end result.’

Participants discussed the influence of the perspectives of others on their vaccine perspectives and how that played out over the course of the pandemic when perspectives diverged. Although this created conflict, many participants were able to come to a place of acceptance (see Figure 2).

When conversations about the vaccine began during the early pandemic, participants primarily discussed the influences of loved ones and family members as they made up their social circle at the time. Many participants received most of their information and general opinions about the vaccine from loved ones: ‘I put a lot of my opinions based on what my mom thinks (…) So, yeah that also influenced a lot of my decisions earlier on.’ Some described how they developed similar views to their loved ones, whether confidence or hesitancy because of conspiracy theories, disbelief or other reasons.

I was with an ex‐partner and he was really against the vaccines. He watched a lot of YouTube videos about it, read a lot about it – about how unhealthy it was – and how unhealthy and bad it was for people to take it and it was going to genetically modify us. So, I was really hesitant because of hearing that almost daily – every day.

Participants in family environments with conflicting opinions discussed how this ‘made it especially even more difficult on whether to take the vaccine or not’. Others who had developed perspectives away from their family and loved ones described disagreement and conflict. Participants felt uncomfortable talking about their perspectives regarding the COVID‐19 vaccine. For some, this led to the avoidance of certain people and conversations.

[W]henever having a conversation with a family member and saying, ‘Oh, you know I'm not too sure if I'll get the vaccine,’ they would immediately question me and be like, ‘Why wouldn't you?’ And it was just – it wasn't a discussion you wanted to have, or you would just re‐questioning yourself (…) So, it's almost like avoidance to have the conversation because I knew what was going to be said and their views.

As the pandemic progressed and society started to reopen, other people's perspectives began affecting youth's perspective. During this time, participants described the impact of their friends and wider social circles. Participants with social groups that had different opinions than loved ones described struggles, disagreements and conflict. One participant noted ‘A lot of the time, for me getting vaccinated meant I would be having arguments with my friends. Because I did not hide that I was vaccinated. I was very proud of the fact that I got vaccinated. So, I shared it. And I – there were a lot of conflicts because of that.’

Some participants described being vaccinated as an eventual ‘acceptance’ or finally ‘giving in’ to the idea that vaccination was inevitable for social integration. A participant recalled ‘I remember before I got the vaccine, I remember all my friends went to Wonderland and I couldn't go because you needed the vaccine. So, I guess also what influenced my decision is the FOMO [fear of missing out].’ Participants discussed the emotional difficulty and conflict, sometimes so severe that it led to them being ‘disowned,’ and they therefore changed their social surroundings.

Many participants reflected on their perspectives in relation to their loved ones and close social circles as the pandemic progressed. They described more positive emotions when society began to open up, allowing themselves to think more clearly and solidify their perspectives, separate from others' influences. Early pandemic conflict gave way to learning to respect others' opinions and ‘agreeing to disagree.’ For example, one participant stated, ‘I had some friends who weren't in the same situation as me and who didn't want to get the vaccine. And it took me a very long time to understand their fears of why they did not want to get the vaccine.’

### 3.3 Complex Societal Influences Affected Views and Decisions Around the COVID‐19 Vaccine

**Figure 3:**
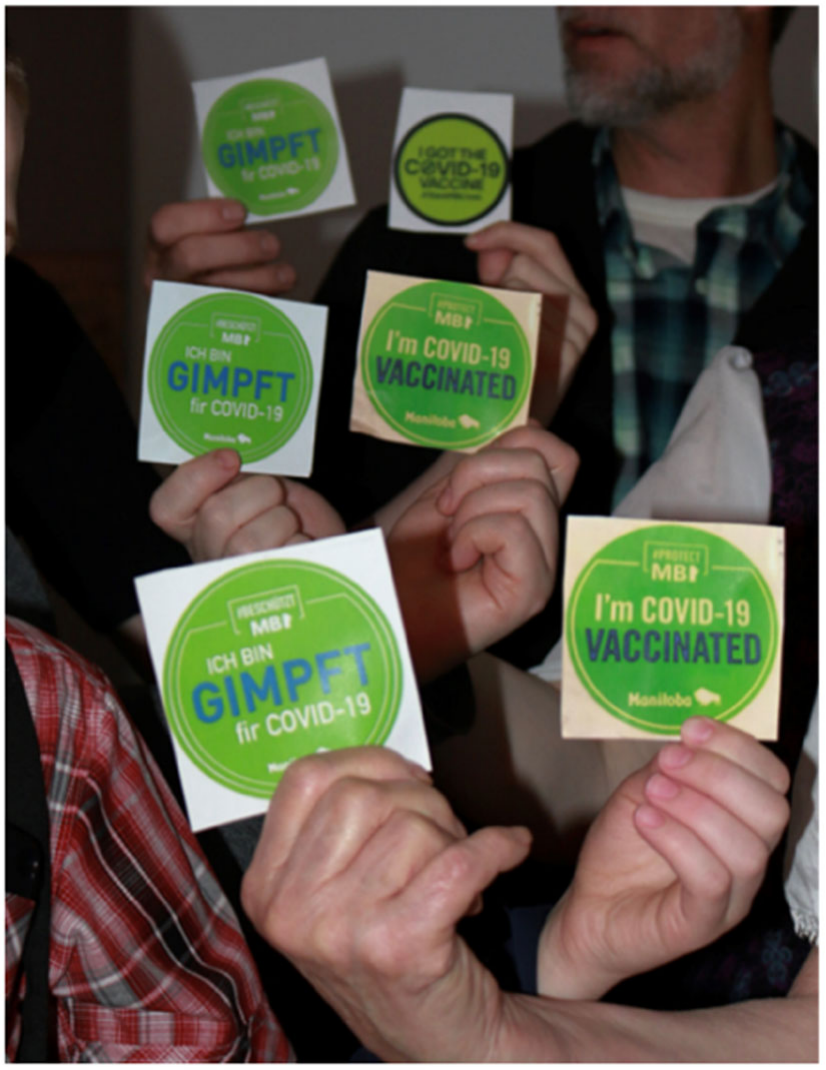
Participant photograph. ‘We are different: In the midst of covid, my community was divided into two opposing sides. Masked vaxxers, and anti‐vax conspiracy theorists. To exist in these harsh times, when one's community is broken, is a difficult thing. Lack of proper communication leads to mistrust and harsh words. I felt alone and isolated as I attempted to make sense of what was happening. Church was closed, communal meals were discontinued, and masks were worn everywhere, hiding familiar faces. This photo represents the two sides trying to reach each other, yet failing, and how I, as a vaccinated person in a mask, felt during those confusing times.’

Participants discussed rigid social norms influencing their perspectives and behaviour towards the vaccine. They recounted their perspectives and willingness to be vaccinated in relation to social aspects and what was viewed as normative behaviour. Society, community and inner social circles influenced participants' journeys with their vaccine perspectives (see Figure 3).

Many participants considered it normative to receive the COVID‐19 vaccine. Participants discussed having a difficult time dealing with the societal view that the COVID‐19 vaccine was the only solution and did not feel as if they were ‘allowed’ to question it, because ‘everyone was assumed and presumably going to get it regardless. (…) It was presumptive.’ Participants felt that the societal ideology did not allow for individual thought or decision‐making. Some participants felt as if society was trying to force and manipulate them into being vaccinated. This led to participants experiencing a battle between preserved societal expectations and their personal perspectives.

I think that also goes back to that hesitancy, because it felt like that individual choice was taken away, in a sense. As soon as someone tells you, you have to do this, I'm like well, now I don't want to do it. So, it's, I don't know, it was just this inner battle where I was going to get vaccinated. But now that you're telling me I have to, I don't want to.

During vaccine development, some participants felt that the idea of a vaccine gave them hope for society's future of returning to normal: it was ‘the light at the end of the tunnel.’ One participant noted ‘I wanted to see the vaccine as something positive – something that could give you a bit of hope; this could all be over soon.’ When the vaccine was released and became a larger part of society, the early inner battle about whether to get vaccinated grew into an inherent pressure to be vaccinated due to government rules and regulations for schools, extracurricular activities and social life.

[I]n order to, like, do a lot of things, like at school to participate in sports, I had to [get vaccinated] anyways. So that's probably why I ended up doing it. So, I could like participate in regular activities.

Participants who were part of the workforce were pressured to choose between their feelings and supporting themselves financially: ‘Ultimately, because of my job I had to. Then it was like I kind of just had to suppress my personal thoughts or my worries. Just not think about it.’ Some participants' experiences with societal pressure led them to make the decision to be vaccinated based on their values and moral reasoning. Participants discussed how the vaccine created sides of ‘good and bad.’ Participants talked about how the wording used in advertisements and the societal expectations around the vaccine perpetuated an almost moral pressure, because ‘if you didn't get the vaccine, you are viewed as a bad person, a rebel and just really shunned by society.’

Societal pressure to be vaccinated also influenced participants' current and future mental health. Participants noted that getting vaccinated would allow them to reenter social life, which was necessary for their mental health. Some felt that it was better to get vaccinated than to deal with the societal consequences of not being vaccinated, including avoiding the negative responses from others, to alleviate their stress and anxiety, as well as needing to be able to participate in social life. One participant explained ‘It almost felt like I was being threatened to either get a vaccine or all of the joys of life will be taken away from me. (…) So, I ended up getting my full two doses, so that I would be able to travel and go to restaurants, because I really needed that for my mental health.’

Participants discussed pro‐social motivations that had a positive effect on their vaccine decisions. They discussed getting vaccinated as a protective measure for themselves, their loved ones, others' loved ones and the health of the country: ‘the empowering, the unity that I felt and the fighting instinct in me that made me get vaccinated. Because I wanted to protect the people that I love.’ Many participants talked about the pro‐social motivations of getting society ‘back to normal’ and getting vaccinated to avoid preventing them from ‘having to live a normal life again.’

As society reopened and vaccine restrictions continued to change or be eliminated, participants reflected on their vaccination decisions. For the participants who felt a lack of autonomy, many stayed hesitant and continued to discuss their disagreement with the mandates.

As the pandemic went on, they started bringing in all these mandates that I completely do not agree with. Stuff like, you cannot go to a restaurant without vaccination (…) you could not go to a park at one point without getting fined. And I just thought that was extremely unfair, and I thought that really took away some of our rights as people. (…) I just really believe that if it's my body, I should be able to do what I want with it, and I feel like everybody should have that and nobody should be able to dictate what I put in my body.

Participants who felt forced to receive the vaccine discussed their ambivalence and/or regret as vaccination requirements were eliminated. Many felt that all the pressure and negative feelings that they experienced were ultimately for nothing. Some said that, in hindsight, they would have considered waiting out the regulations and restrictions instead of being vaccinated: ‘I don't think it was personally the right choice for me.’

Participants who discussed getting vaccinated due to pro‐social motivations talked about how the vaccine was a positive and/or necessary factor for the progression of society. They felt proud and empowered about their decision to be vaccinated. They also believed that without the vaccine, the regulations and restrictions probably would have needed to stay in place. One participant stated ‘I think it kind of did help us progress as a society.’

### 3.4 Youth Navigated Their Vaccine Journeys Through First‐ and Second‐Hand Experiences

**Figure 4:**
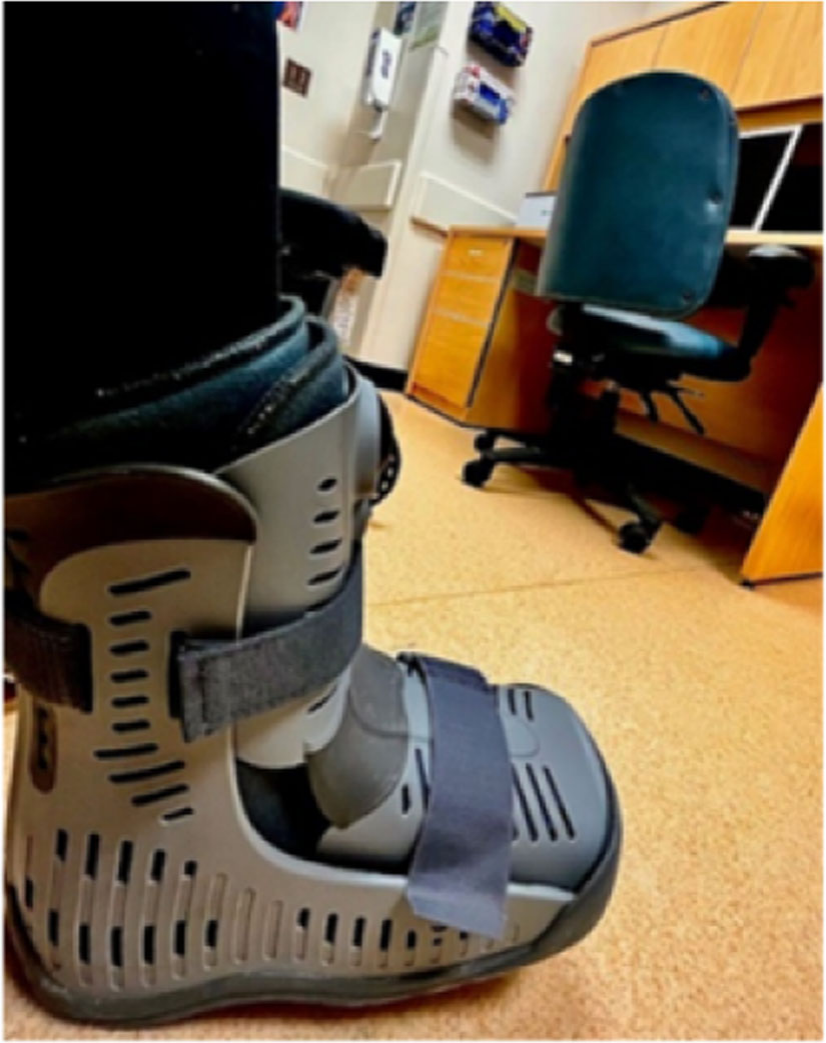
Participant photograph. ‘My walking boot at my initial appointment, if I didn't trust the science I wouldn't be as on the road to recovery as I am now‐ and that's why I have confidence in the Covid‐19 vaccines.’

The final theme highlights the influence of first‐ and second‐hand experiences on vaccine perspectives through the course of the pandemic. Past experiences with medicine and the healthcare system shaped early opinions, but opinions evolved as participants gained direct experience with COVID‐19 and the COVID‐19 vaccines (see Figure 4).

Participants analysed how early first‐ or second‐hand pandemic experiences with healthcare affected their vaccine perspectives. Negative past experiences within healthcare built ‘that lack of trust in the healthcare system’.

In contrast, participants who had had previous positive experiences with healthcare talked about having trust in the medical system and more confidence in the vaccine: 'I put a lot of faith in the healthcare system with my ankle, and that's why you put a lot of faith into the healthcare system with the vaccines.' As the COVID‐19 vaccines rolled out, many participants noted that witnessing loved ones and others receive the vaccine had a positive impact on their perspective of the vaccine.

I know what else impacted my decision was waiting a bit to see if the vaccine was safe in a way. Like, seeing other people take it. (…) I waited for my mom, I mean not my mom, my sister, and my dad to take it first. And that helped me become more confident with it because people that like, that I love and look up to, they took the vaccine, so yeah that's primarily it.

Some participants discussed how having positive experiences with healthcare during this time helped them develop trust in the medical system and ease their vaccine hesitancy. These types of first‐ and second‐hand experiences included vaccine appointments, hospital visits, doctor appointments and so forth: ‘Because, like, actually having to experience that, it showed me that there's like nothing to be afraid of.’

Participants who experienced or witnessed COVID‐19 infections with versus without the vaccine gained an enhanced understanding of the vaccine's efficacy and developed vaccine confidence.

I guess for me, the main reason that I find confidence is because every single member in my family, except my mother, like everyone, my cousins, my aunts, my distant cousins, they all have had vaccines, and they all are fine. And their reactions to actually getting COVID was very minor. And my mom, on the other hand, who, obviously, when she got COVID, it was really, really, really bad. And I think that was proof for me that the vaccines do help a lot.

However, positive experiences were not universal: ‘Every time I got COVID, my COVID would get worse. And they kept saying, “Oh, it only reduces the severity of symptoms,” but that's the complete opposite of what I actually experienced.’

As the pandemic progressed and life started to return to normal, participants reflected on their early pandemic experiences and the way they influenced their late‐pandemic perspectives. For some participants, despite being vaccinated, their distrust towards healthcare and the vaccine reemerged or was never resolved. For other participants, the experience of those or people around them who became extremely ill from COVID‐19 and those who experienced side effects from the vaccine elicited a resurgence of hesitancy or an increase in mistrust towards the healthcare system.

So, yes, the hesitancy with vaccines kind of grew on me. I wasn't scared of the vaccines, because in general, I haven't had any problems with vaccines. But what I find with this vaccine in particular, and from what I've seen is that it doesn't work. There's side‐effects that are quite severe, and I don't think it was well researched enough before they released it to the general public.

## 4 Discussion

This study examined the COVID‐19 vaccine perspectives of Canadian youth with MHSU challenges using a participatory research methodology of photovoice. Four main themes described youth journeys with the COVID‐19 vaccine perspective, encompassing the changing information landscape, mixed perspectives among social and familial structures, a variety of complex social influences, and the impact of first‐ and second‐hand experiences. The four themes and subthemes highlight the evolution of participants' journeys with the COVID‐19 vaccine from the early pandemic through to their late‐pandemic perspectives.

Youth COVID‐19 vaccine perspectives were largely impacted by social and societal influences, as demonstrated in previous research. Social circles can have both positive and negative impacts [47, 48]. For example, when social circles espouse ‘pro‐vaccine’ views and behaviours, this encourages vaccination and vaccine acceptance in youth [49, 50]. However, when social circles have conflicting views, conflict between family, friends and/or social groups can occur [51]. Likewise, social norms can be powerful motivators. Social norms influenced the uptake of the COVID‐19 vaccine, including the social responsibility to protect others [52, 53] and a moral obligation to return to normal life [51]. However, perceived or internalized pressure negatively affects mental health and impacts levels of vaccine hesitancy [54]. This supports the WHO's determinants of vaccine hesitancy and the ‘3 Cs’ can be affected by individual, group and contextual influences [20]. Mandating vaccination by imposing restrictions on social life can promote attitudes of violated human rights, stigma, distrust, social polarization and adverse effects on mental health and well‐being [55], including among youth.

Lack of confidence or trust in the system was discussed through participants' first‐ and second‐hand experiences. Mistrust in vaccines has been linked to past experiences with the healthcare system [53]. Socially and economically marginalized groups typically experience ongoing systematic injustices, discrimination and lack of access and are more likely to have medical mistrust [56]. A lack of trust in health institutions influences the fear of vaccine development, side effects, future effects and conspiracy‐related beliefs [57, 58]. Previous research on other vaccines has similarly found that vaccination decisions can be based on personal first‐ and second‐hand experiences, perceptions of health and a wide range of personal and experiential factors [59]. Inaccurate information [57], unreliable sources [60], information overload [61], rumours and myths [62] all have the potential to build mistrust in vaccines. A lack of confidence in government decision‐making on COVID‐19‐related issues and vaccine promotion fosters distrust in governmental institutions and facilitates doubt about the vaccine [51, 57, 63]. Establishing confidence in the medical and healthcare system is a key component of the WHO ‘3 Cs’ and involves a wide range of economic, historical, societal and psychological factors [20, 64]. Informed, effective vaccine uptake strategies for youth should be built with an awareness of the importance of trust and mistrust not only in the vaccine, but also in the healthcare system as a whole.

Our results show that youth COVID‐19 vaccine perspectives varied through the pandemic. Previous longitudinal research has found that vaccine hesitancy can remain the same [65], steadily decrease or increase [66], rise and fall [67] and even change as rapidly as week to week [68]. Research has also examined a rise in vaccine hesitancy and a decrease in the intention to be vaccinated with the COVID‐19 vaccine after it became available [67, 69, 70]. The emergence of booster shots may have fuelled a resurgence of vaccine hesitancy [71]. This suggests that timing may be an important factor to consider in terms of building youth vaccine confidence.

Our participants discussed the importance that information and vaccine communication had on their COVID‐19 vaccine perspectives. Encountering a wide range of misinformation about the COVID‐19 vaccine resulted in confusion, distress, mistrust and a less positive perspective of the vaccine [45]. Other research has also suggested the importance that emotion plays in COVID‐19 vaccine communication, highlighting how both negative and positive emotions may be leveraged in vaccine hesitancy and confidence [72]. Previous research supports that individuals found it difficult to debunk false information and make informed decisions due to contradictory messages from leaders, the quantity of false information on social media and the complexity of government and media messaging [60]. Believing that the COVID‐19 vaccine was unsafe, knowing less about the vaccine and being more likely to believe myths have been associated with lower education and income [73]. Our findings have supported the research suggesting that the WHO ‘3 Cs’, should include a fourth ‘C’ that refers to communication [21].

Vaccine decisions are more complex than simple binary decisions. Vaccine confidence is not a linear experience, and youth are still at risk of experiencing hesitancy. Notably, our findings show that hesitancy does not always directly relate to vaccine uptake, as many of our participants considered themselves hesitant but still had some or all vaccines. Several public health interventions focus on education and the promotion of accurate information to encourage vaccination and decrease vaccine hesitancy [74, 75, 76, 77]. In line with previous research, our findings indicate that although information is an important component, vaccination campaigns should also incorporate broader sociocultural aspects, including awareness of the influences of social circles, social conflict, societal norms, present and past experiences, the emotional burden of the decision and the influence of time. This aligns with the newly proposed ‘4 Cs’ model that describes the complexities of vaccine hesitancy and its determinants across a broad and encompassing number of actionable factors [20].

The strengths of this research project include youth leadership, youth and lived experience engagement, strong retention, and a sizable and relatively diverse sample. However, certain subgroups of youth were underrepresented, which made it impossible to arrive at specific conclusions regarding differences based on ethnicity, gender and other personal characteristics. The recruitment materials may have specifically attracted youth with an interest in photography. The project was conducted virtually, and therefore, the sample was limited to participants who had regular and stable access to photographic equipment, the internet and a computer. Future research should be conducted with youth who are not regular Internet users or who have limited access to the Internet.

Youth with MHSU challenges navigated a complex environment during the COVID‐19 pandemic, as they were faced with the decision about whether to be vaccinated against the virus. A wide variety of factors influenced their decisions, including the rapidly changing informational landscape, the conflicting perspectives of people in their social circles, the rigid societal norms and their past and emerging experiences of health, healthcare, COVID‐19 and the COVID‐19 vaccine. The wide variety of factors influencing vaccine perspectives should be taken into account in public health messaging and future research on youth vaccine uptake. Youth‐led and youth‐engaged research can help solicit rich and meaningful perspectives of young people on important public health issues.

## Author Contributions

**Shelby Mckee:** methodology, investigation, formal analysis, writing–original draft, and writing–review and editing. **Natasha Y. Sheikhan:** conceptualization, methodology, formal analysis, writing–review and editing, and funding acquisition. **Sean Patenaude:** conceptualization, funding acquisition, writing–review and editing, and investigation. **Jo Henderson:** conceptualization, methodology, writing–review and editing, resources, and funding acquisition. **Rodney Knight:** conceptualization, methodology, writing–review and editing, resources, and funding acquisition. **Sean A. Kidd:** conceptualization, methodology, writing–review and editing, and funding acquisition. **Skye Barbic:** conceptualization, methodology, writing–review and editing, and funding acquisition. **Aileen O'Reilly:** conceptualization, methodology, writing–review and editing, and funding acquisition. **Lisa D. Hawke:** conceptualization, investigation, funding acquisition, writing–review and editing, supervision, project administration, resources, formal analysis, and methodology.

## Ethics Statement

Ethics approval was obtained from the Research Ethics Board of the Centre for Addiction and Mental Health (#155‐2021).

## Conflicts of Interest

The authors declare no conflicts of interest.

## Supporting information

Supporting information.

## Data Availability

The final photographs submitted as part of this project are publicly available at https://www.camh.ca/en/science-and-research/research-connect/youth-perspective-on-covid-19-vaccine/photovoice-project. The other data for this project are governed by the Research Ethics Board of the Centre for Addiction and Mental Health. Any requests for access should be made to the corresponding author.
