# Supplementary material for: ‘Is It Safe? Is it not?’ A Youth‐Led Photovoice Study of Youth Perspectives of COVID‐19 Vaccine Confidence
Source: Health Expect. 2024 Oct 5;27(5):e70051. doi: 10.1111/hex.70051 (PMC11456145; doi:10.1111/hex.70051)
Supplement: Supplementary file 1 — Supporting information. [file HEX-27-e70051-s001.docx]

**APPENDIX A**

**Semi-Structured Interview guide**

1. **Review of Informed Consent and interview structure**

This session will be audio recorded and will last about 2 hours (or 120 minutes). As you remember, from the up to seven workshops you attended, we asked you to take photographs of your experience and personal perspective on COVID-19 vaccine confidence. Today, we’d like to talk about what your photos say about vaccine confidence. You’ve all worked very hard to produce amazing artwork. Now is the time to tell us what it means to you. We’ll begin today with each member showing the photo or a few photos, that are the most meaningful to them and telling us a bit about them. Then we will ask you some questions about vaccine confidence. During the interview, I’ll be taking a few notes about the events and experiences you describe to me.

Do you have any questions about how we’re going to spend our time today?

1. **Introductions & Icebreaker**

[Each person goes around and introduces themselves & ice breaker question activity]

Before we get started with talking about your photography and asking questions about your experience with COVID-19 vaccines. We want to acknowledge that there are no wrong answers. All opinions, feelings, and perspectives are valid and welcomed. We are here today to learn and have an open, non-judgmental conversation about photography and vaccines.

1. **Review of Photographs**

We would now like to review your photographs one by one. To the best of your ability, please describe what you were trying to capture with each one and the meaning behind them.

- - ***Probe:*** Tell me what this picture is about
  - ***Probe:*** What were you trying to say within this image that is hard to say with words?
  - ***Probe:*** If this photograph was part of an exhibit, what would you want the viewers to know about the photograph?
  - ***Probe:*** How do you think your mental health impacted your photography?

Now we will go around and talk about each other’s photography.

- What about the rest of the group? What do you see when you look at this photo?
- What is one or a few words that comes to mind when you look at these photographs?
  - ***Probe:*** What does that word mean to you?
- What does this photograph tell you? How does it tell you that?
- What do you think other youth will think about when they look at this?

1. **COVID-19 vaccine experience**

Now we would like to talk about your experience with vaccine hesitancy.

- What has been your journey with the vaccine?
- Why do you think you were hesitant about COVID-19 vaccines?
  - ***Probe:*** Mistrust? Unknown health concerns? Misinformation? Lack of Clear, Consistent, Youth-Focused Messaging? Accessibility challenges? Communal reinforcement?
  - ***Probe*:** How do people around you feel about the vaccine?
  - ***Probe:*** How do you think your mental health impacted your confidence about the vaccine?
- How has your perspective of the vaccine changed over time?
  - ***Probe:*** When it first came out? After hearing about numerous boosters? After completing the project?
  - ***Probe***: Confident to hesitant? Hesitant to confident? Somewhere in the middle?
  - ***Probe***: Has it been dynamic? Were there ebbs and flows?
  - ***Probe***: How do you think your mental health impacted your change in perspective?
- What does vaccine hesitancy mean to all of you?
- What does vaccine confidence mean to you?
- How did the process of taking photos affect your perspective?
- Let’s pretend for a second that next year we were to have another pandemic. What steps do you think we could take to foster vaccine confidence within the community?
  - ***Probe:*** What things should we avoid?

1. **Experience with the project**

The next set of questions is to elaborate on your experience with this project and what you’d like people to know

- Why was it important to tell the story this way?
- What was special about using photography to take about this topic?
- Why should people care about this project?
- Generally speaking, how do you think youth approach the vaccine?
- How has this project affected the way you feel about the vaccine?

1. **Final Questions**

- If you can reflect back on everything we talked about today, what is one thing you would want policy markers to take from today?
- Do you have anything else you want to tell us about COVID-19 vaccine confidence?

[Thank the participants, conclude the focus group].
